# Device for Measuring Contact Reaction Forces during Animal Adhesion Landing/Takeoff from Leaf-like Compliant Substrates

**DOI:** 10.3390/biomimetics9030141

**Published:** 2024-02-26

**Authors:** Zhouyi Wang, Yiping Feng, Bingcheng Wang, Jiwei Yuan, Baowen Zhang, Yi Song, Xuan Wu, Lei Li, Weipeng Li, Zhendong Dai

**Affiliations:** 1 College of Mechanical and Electrical Engineering, Nanjing University of Aeronautics and Astronautics, Nanjing 210016, China; fengpan37@outlook.com (Y.F.); bingcheng.wang@uzh.ch (B.W.); jiweiyuan@nuaa.edu.cn (J.Y.); zbw123456@nuaa.edu.cn (B.Z.); zddai@nuaa.edu.cn (Z.D.); 2 Nanjing University of Aeronautics and Astronautics Shenzhen Research Institute, Shenzhen 518063, China; 3 Institute of Neuroinformatics, University of Zurich and ETH Zurich, 8057 Zurich, Switzerland; 4 College of Mechanical Engineering, Zhejiang University of Technology, 18 Chaowang Road, Hangzhou 310014, China; 5 Robotics Laboratory China Nanhu Academy of Electronics and Information Technology, Jiaxing 314000, China; wuxuan@cnaeit.com (X.W.); lilei@cnaeit.com (L.L.); liweipeng@cnaeit.com (W.L.)

**Keywords:** leaf-like compliant substrate, contact reaction force, force measurement device, landing, takeoff

## Abstract

A precise measurement of animal behavior and reaction forces from their surroundings can help elucidate the fundamental principle of animal locomotion, such as landing and takeoff. Compared with stiff substrates, compliant substrates, like leaves, readily yield to loads, presenting grand challenges in measuring the reaction forces on the substrates involving compliance. To gain insight into the kinematic mechanisms and structural–functional evolution associated with arboreal animal locomotion, this study introduces an innovative device that facilitates the quantification of the reaction forces on compliant substrates, like leaves. By utilizing the stiffness–damping characteristics of servomotors and the adjustable length of a cantilever structure, the substrate compliance of the device can be accurately controlled. The substrate was further connected to a force sensor and an acceleration sensor. With the cooperation of these sensors, the measured interaction force between the animal and the compliant substrate prevented the effects of inertial force coupling. The device was calibrated under preset conditions, and its force measurement accuracy was validated, with the error between the actual measured and theoretical values being no greater than 10%. Force curves were measured, and frictional adhesion coefficients were calculated from comparative experiments on the landing/takeoff of adherent animals (tree frogs and geckos) on this device. Analysis revealed that the adhesion force limits were significantly lower than previously reported values (0.2~0.4 times those estimated in previous research). This apparatus provides mechanical evidence for elucidating structural–functional relationships exhibited by animals during locomotion and can serve as an experimental platform for optimizing the locomotion of bioinspired robots on compliant substrates.

## 1. Introduction

**Figure 1:**
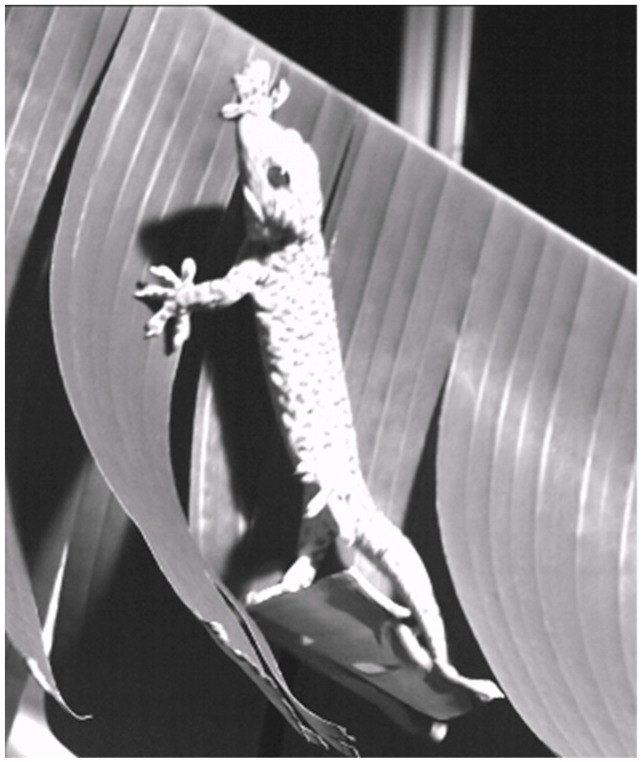
Animal locomotion on compliant substrates such as foliage. The landing behavior of the *Gekko gecko* on foliage.

The living environments of animals are complex, necessitating continual adaptation to a diverse array of terrains and substrates for survival. Many animals have evolved remarkable locomotive capabilities in response to intricate substrate environments. In terrestrial creatures, in addition to common solid and stable substrates, such as rocks and tree trunks, compliant substrates, such as leaves and soft branches, also exist [1,2,3]. Such substrate environments are prevalent in the upper reaches of tree canopies, where numerous arboreal species are frequently active [4,5,6]. The flexibility and effectiveness of animal movements are influenced by the compliance of the substrate [7,8,9], as high-compliance substrates introduce factors affecting energy loss and movement posture disturbances during locomotion [10,11,12]. Studies have revealed a correlation between locomotive capabilities and preferences of animals (such as lizards and primates) and substrate compliance [13,14,15]. Challenges posed by takeoff and landing are particularly difficult for animals adept at arboreal habitats [10,16,17]. Different species exhibit distinct strategies; for instance, during the takeoff leap, primates and lizards complete their jumping motion before the compliant substrate rebounds [11,12,18], thereby losing the energy stored in the compliant substrate. In contrast, Cuban tree frogs can leave the substrate after rebounding, thereby recovering a portion of the energy stored in the compliant substrate [19]. Accidental falls due to compliance-induced instability in higher ecological niches may have severe consequences [20]. During rapid descent on flexible rods, frogs exhibit high-performance dynamic friction effects, whereas the high friction adhesion capabilities of geckos may contribute to their successful landing on compliant substrates [16]. Additionally, we captured geckos’ landing behavior on real flexible leaves in laboratory settings (Figure 1). However, further adhesive contact force testing is required to validate these claims and analyze related phenomena in depth.

The differences in behavioral strategies when faced with high-compliance substrates imply variations in the underlying mechanisms of animal motion, particularly in how animals ensure sufficient adhesion while unloading impact forces during the moment of contact with highly deformable substrates. Conversely, how do adhesive animals instantaneously release adhesive forces and generate sufficient propulsive forces to takeoff from the substrate? Existing research on the functional aspects of animal movement on compliant substrates has primarily focused on the theoretical modeling and analysis of locomotor behavior [21,22,23], as well as partial energy analyses and electromyographic signal studies [10,24,25]. This concentration is largely due to the lack of an effective simulation of compliant substrates and precise measurement of contact forces. Different compliant substrates have specific characteristics of low stiffness and damping attenuation and are easy to yield to external loads. So, measuring the reaction force on compliant substrates contains great challenges. Consequently, to enhance our understanding of the biomechanics and structural–functional evolution of locomotion, especially in the context of adhesive movements, the development of novel devices is imperative to enable the effective simulation of large compliant substrates and to conduct measurements of the mechanics of contact forces.

In existing studies that focus on the simulation of compliant substrates, a well-established method uses the combination of rigid and elastic components to approximate the simulation of flexible structures. In early research on primates, a vertical rod with a certain flexibility was employed as a substrate, and the jumping and landing forces of primates were inferred from the strain on the rod [26]. Subsequently, researchers installed spring devices beneath rigid plates and concentrated on characterizing the low-stiffness properties of the substrate through these spring devices [11], rendering the low-stiffness characteristics of the substrate more controllable. When studying the motion of Cuban tree frogs on compliant substrates, researchers employed servomotors to centrally simulate the stiffness characteristics of the substrate [10], further enhancing the controllability of substrate stiffness. However, because most elastic components exhibit damping characteristics, the ability to control damping characteristics is indispensable for the simulation of compliant substrates because damping characteristics can affect the simulation accuracy of substrate compliance in terms of response speed, response accuracy, and other aspects. Moreover, damping characteristics are often unknown, emphasizing the necessity of introducing controllable damping characteristics to simulate compliant substrates. Therefore, in order to enhance the accuracy of simulating the target substrate, the new force measurement device not only needs to control the stiffness characteristics for compliant substrates but also requires the introduction of control over damping characteristics.

Furthermore, in mechanical measurements targeting high-compliance substrates, the available force measurement devices are primarily of two types: rigid and flexible sensors. With continuous advancements in sensor technology, strain-based sensor technologies designed for various complex rigid measurement scenarios have become increasingly mature [27,28,29]. However, owing to constraints such as precision, material properties, and dynamic properties associated with flexible sensors [30,31,32], current research outcomes in compliant measurements primarily rely on equivalent measurements of contact mechanics through traditional rigid strain-based sensors [11,33,34]. The reaction force between the animal and the substrate requires the presence of contact. While facing the compliant substrate, the force signal measured by strain sensors and its principles still shows large numerical fluctuations after the contact is removed [26]. This phenomenon may be due to the lack of effective measurement of the oscillatory inertial force of the compliant substrate under the impact, leading to the coupling of the oscillatory inertial force of the substrate in the contact force results, which restricts the contact force measurement accuracy of the compliant substrates. In addition, some research results mainly focus on measuring and analyzing normal contact force [10,26]. However, for adhesive animals, the tangential contact force with the substrate is also crucial for their movement [16,35]. In order to improve the accuracy of force measurement on compliant substrates, new force measurement devices should take the inertial force due to the substrate oscillation into account and be capable of measuring the tangential contact force.

In light of the aforementioned analysis, the new force measurement device designed for compliant substrate environments should exhibit the following characteristics: the capability to control both stiffness and damping properties when simulating compliant substrates and enable real-time measurement of inertial forces while encompassing both normal and tangential directions of contact forces. In response to these challenges, this study introduces a novel device designed for kinematic testing of locomotion on compliant substrates. The device employs servomotors instead of elastic elements to simulate the stiffness and damping characteristics of the compliant substrates. Real-time and precise measurements of contact forces between animals and substrates are facilitated by the synchronous measurement of multidimensional forces and acceleration sensors. Calibration of the device for adhesive contact was achieved through experiments using non-adhesive and adhesive standard blocks. Comparative analyses of the jumping/landing behaviors of adhesive animals in conjunction with non-adhesive/adhesive standard block experiments were conducted to validate the testing performance of the device. The development of this device is expected to advance our understanding of the biomechanics and evolutionary structural and functional aspects of animal adhesion movements. This provides a novel research method and tool for studies in fields such as ethology, biomechanics, and neuroscience. Consequently, it inspires and facilitates developments in the structural design and control strategies of bioinspired robots, thereby contributing to the progress of biomimetic robotics.

## 2. Materials and Methods

### 2.1. Components of the Device

**Figure 2:**
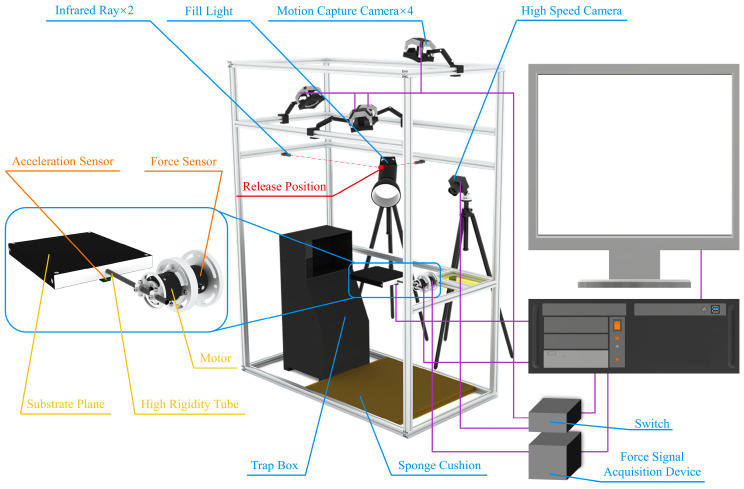
Leaf-like compliant substrate measurement device. This setup primarily comprises leaf-like compliant substrates, a contact mechanics measurement system, motion capture cameras, high-speed cameras, a central control system, and a lure box. The leaf-like compliant substrates and contact mechanics testing system integrate multidimensional force sensors, acceleration sensors, motors, as well as a base plane and a high-stiffness carbon fiber tube.

This study focused on the locomotion behaviors and mechanics of animals landing or jumping on compliant substrates, such as large leaves and slender branches. A specialized experimental device for investigating the biomechanics of animal takeoff and landing on compliant substrates (Figure 2) was developed. Oscillation parameters simulating the primary vein oscillation of leaves were employed as an example. The adjustment of these parameters could also simulate and characterize the damping oscillations on compliant substrates that resemble slender branches. The device primarily consists of leaf-like compliant substrates, a contact mechanics measurement system, a motion behavior capture system, and various experimental auxiliary devices. The control of each system is integrated into a central control system to achieve synchronized operations through a coordinated program.

A leaf-like compliant substrate was employed to emulate the bending oscillation characteristics of leaf veins at various points along the main vein under the influence of applied loads. It primarily consisted of a torque motor, connecting rods, and a load-bearing plate. The connecting rod and load-bearing plate were affixed to the servomotor using lightweight, highly rigid aluminum alloy clamps. The motor was connected to the base force sensor system by a flange clamp. The connecting rod and load-bearing plate must possess high rigidity and lightweight characteristics, enabling concentrated emulation and control of the damping-compliant oscillation properties at points simulating the primary leaf vein on the compliant substrate. This design also circumvents the risk of expression failure in compliant features owing to an excessively low animal–substrate mass ratio and ensures the reliability of the load-bearing module within the simulation system. Consequently, carbon fiber tubes (T300, Shenzhen Nuodi combined material Ltd. Shenzhen, China), known for their higher stiffness, were employed as the connecting rod, whereas high-density foam boards (18 K) served as the load-bearing plate, resulting in a combined mass of only 184.2 g. Typically, the movement frequency of adhesive crawling animals does not exceed 10 Hz [36]. To prevent simulation distortion of the substrate’s damping oscillation compliance characteristics concerning leaf veins, a ^TM^ DM-J4310-2EC reduction motor (outer diameter 56 mm, height 46 mm) was selected, with an actual tested control frequency of 170.54 ± 0.14 Hz (*n* = 10), meeting the experimental requirements.

A contact mechanics measurement system and motion behavior capture system were employed to record the contact forces and behavioral changes of the animal adhesion landing or takeoff from the compliant substrate. The contact mechanics measurement system comprised a multidimensional force sensor (HKM-MIOS-Y60-H50, Anhui ZHONGKE MI POINT Sensor Co., Ltd., Heifei, China, 2000 Hz, outer diameter 60 mm, height 50 mm), an IMU module (JY931, WIT Ltd.,Nanjing, China, 500 Hz), and a data acquisition card (NI-9237). The sampling rate of each sensor was not less than 300 Hz. Synchronized control programs from the central control system enabled the coordinated measurement of contact forces and behaviors between the contact mechanics measurement system and motion capture system. The motion capture system comprised four motion capture cameras (Prime 17 W, OptiTrack Ltd., Corvallis, OR, USA, FPS = 360 Hz) and a high-speed camera (QianYanLang ISP502, ZhongKeShiJie, Heifei, China, 800 FPS).

In addition to the aforementioned primary components, this device is also equipped with auxiliary experimental devices, including an infrared emitter to ensure release positioning, an animal trapping box to create a dark environment conducive to animal adaptation, and sponge cushioning pads to prevent injury to the animals. These apparatuses primarily aim to ensure experimental precision, facilitate animal release, and ensure the safety of animals during experimentation.

### 2.2. Simulation of Compliant Substrates

**Figure 3:**
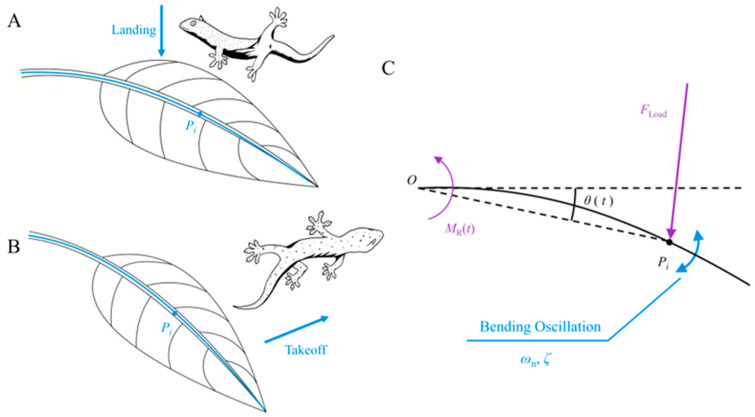
Animal landing and jumping behavior on leaf substrates. (**A**,**B**) depict the process of a gecko landing on and leaping from a leaf, respectively. Point $P_{i}$ represents the landing and takeoff positions, corresponding to the simulated points on the target substrate. (**C**) provides an analytical diagram of the leaf’s loading response process, where $F_{\mathrm{Load}}$ and $M_{R}(t)$ represent the external load and the resistance torque generated during substrate loading, respectively. $\omega_{n}$ and ζ denote the undamped natural frequency and damping ratio of the angular swing at point $P_{i}$.

In this study, a simulation of a large leaf-like compliant substrate (analogous to the investigation of fine branches) is employed as an illustrative case (Figure 3A,B). Our primary focus, as in previous studies [16], is to study the oscillatory responses induced by animal locomotion along the bending direction of the leaf (Figure 3C). Although an intricate diversity of responses is inherent in leaf-like substrates [37,38,39,40,41], our objective is not to precisely replicate every nuanced response across the leaf surface. The loaded motion process along the bending direction of leaves within a limited number of damping periods is treated as a second-order system response process [18,42]. Consequently, the temporal evolution characteristics of the loaded motion at point *P_i_* along the main leaf vein in the bending direction of the leaf can be described by the simplified “Mass-damping-stiffness” form of vibration Equation (1) [42] as follows:

(1) $$J\ddot{\theta }(t)+D\dot{\theta }(t)+K[\theta (t)-\theta_{0}]=M_{\mathrm{Load}}(t)$$

where *J* represents the rotational inertia around the rotational center (base of the leaf stem), $\theta_{0}$ is the initial angle of the compliant substrate, and θ(t), $\dot{\theta }(t)$, and $\ddot{\theta }(t)$, respectively, denote the functions of the angle, angular velocity, and angular acceleration of the compliant substrate with respect to time *t*. *D* denotes the damping in the bending oscillation of the substrate, and *K* represents the bending stiffness of the compliant substrate. Additionally [42],

(2) $$K=J\omega_{n}^{2},\;D=2J\zeta \omega_{n}$$

where $\omega_{n}$ represents the undamped natural frequency of the bending motion of the compliant substrate, and ζ is the damping ratio of the bending motion of the compliant substrate. The left side of Equation (1) can be divided into two components constituting the inertial torque and resistance torque as follows:

(3) $$M_{I}(t)+M_{R}(t)=M_{\mathrm{Load}}(t).$$

where $M_{\mathrm{Load}}(t)$ represents the externally applied load torque, $M_{R}(t)$ originates from the resistance torque induced by the stiffness–damping characteristics of the compliant substrate, and $M_{I}(t)$ stems from the inertial torque associated with the rotational inertia of the compliant substrate. In this study, an actively controllable motor was employed to adjust the resistance torque of the compliant substrate, denoted as $M_{R}$, in lieu of conventional passive response elastic elements to precisely emulate the target compliance characteristics. This approach allows not only the adjustment of the stiffness of the system but also significantly enhances the accuracy of simulating the compliance characteristics of the target substrate by controlling the damping. Therefore, the control model for the motor is expressed as follows:

(4) $$M_{R}(t)=2J\zeta \omega_{n}\dot{\theta }(t)+J\omega_{n}^{2}[\theta (t)-\theta_{0}]$$

where the undamped natural frequency $\omega_{n}$ and damping ratio ζ of the leaf-like compliant substrate are determined based on the characteristic parameters required for simulating the target. $J_{R}$ represents the rotational inertia of the leaf-like compliant substrate. This implies that by adjusting the system’s output stiffness, damping characteristics, and cantilever length, the loaded compliant motion characteristics of various main leaf vein points $P_{i}$ (target substrates) can be emulated.

In this study, referring to the habitats of adhesive animals and previous research [1], palm leaves were chosen as the target substrate for constructing a leaf-like compliant substrate. Five randomly selected fresh palm leaves were used, and a uniform distance of 95 cm from the clamping position along the leaf vein was designated as point $P_{i}$. Employing the free decay method [18], a total of 75 experiments were conducted, revealing an intrinsic frequency distribution in the range of 5.81–6.50 (*n* = 75) and a damping ratio distribution in the range of 0.053–0.087 (*n* = 75) for this batch of palm leaves. Consequently, the selection range for the simulation control parameters of the motor was clarified. To further investigate the influence of a compliant substrate on animal locomotion, the lower bounds of the undamped natural frequency and damping ratio of the target leaf were chosen as control parameters to simulate a compliant substrate with $\omega_{n}=5.81$ and ζ=0.053 to manifest heightened compliance.

The performance characteristics of the device must be calibrated through experiments to eliminate or reduce the influence of uncertain factors, such as air resistance and mechanical friction. First, the actual rotational inertia $J_{a}$ of the simulated substrate must be calibrated. As the components employed in the experimental setup are characterized by high stiffness, the substrate stiffness is determined by the natural frequency and rotational inertia of the system (Equation (2)). The inherent physical properties of the system’s stiffness can be utilized to calibrate the rotational inertia of the rotational part. This was achieved by predefining a set of rotational inertias and undamped natural frequencies ($J_{\mathrm{apx}}$ and $\omega_{\mathrm{apx}}$) in the control program corresponding to the simulated stiffness K. Using the free decay method, the compliant substrate was induced to undergo free oscillations, and the motion capture system recorded the oscillation trajectory. The actual undamped natural frequency $\omega_{a}$ under the simulated stiffness K was obtained, from which, using Equation (5), the actual rotational inertia of the device $J_{a}$ was calculated (mean = 0.0274 $\mathrm{kg}\cdot m^{2}$, *n* = 16).

(5) $$K=J_{\mathrm{apx}}\omega_{\mathrm{apx}}^{2}=J_{a}\omega_{a}^{2}$$

where $J_{\mathrm{apx}}$ represents the preset value of rotational inertia, $\omega_{\mathrm{apx}}$ is the predefined undamped natural frequency in the program, and $\omega_{a}$ denotes the actual undamped natural frequency of the oscillation. Although the calibrated values of rotational inertia were consistently used in the subsequent experiments in this study, altering the length of the cantilever structure allows for the adjustment of the rotational inertia and thereby its specific numerical calibration via the aforementioned process.

Subject to the damping effects inherent in the motor itself and external factors, such as air resistance, it is necessary to adjust the damping parameters. After obtaining the actual rotational inertia $J_{a}$, the target damping ratio $\zeta_{\mathrm{apx}}$ was set in the program, and a calibration experiment was conducted using the free decay method to determine the actual damping ratio $\zeta_{a}$. The corrective value $\zeta_{d}$ for the damping ratio was computed using Equation (6).

(6) $$\zeta_{d}=\zeta_{a}-\zeta_{\mathrm{apx}}$$

The corrective value $\zeta_{d}$ was input into the simulation program to compensate for the impact of unknown damping factors.

**Figure 4:**
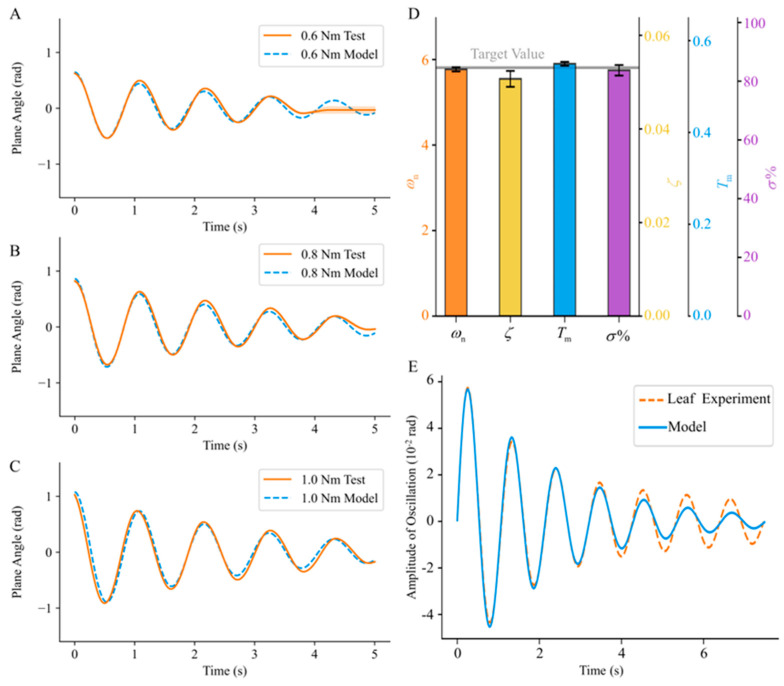
Calibration results of target base motion simulation. (**A**–**C**) depict the free decay oscillation angle curves under three initial torque loads of 0.6, 0.8, and 1.0 Nm, respectively. The orange solid lines represent the actual experimental results, whereas the blue dashed lines represent the theoretical simulation model. (**D**) illustrates the results of four parameter indicators, namely, undamped natural frequency $\omega_{n}$, damping ratio ζ, peak time $T_{m}$, and maximum overshoot σ%, after calibration, with gray horizontal lines indicating the preset target values for each parameter. (**E**) presents a comparison between the experimentally measured free decay curve of the leaf and the simulation model. The alignment is particularly pronounced in the first 3 to 4 decay cycles.

Following the aforementioned calibration of the intrinsic parameters of the device, free decay motions were conducted under three initial loads: 0.6, 0.8, and 1.0 Nm. A comparative analysis was performed between the actual decay curves obtained during the experimental process and the control curves to evaluate the degree of conformity between the simulated response of the device and the theoretical model. The results are shown in Figure 4A–C. Regarding the initial response simulated on the compliant substrate, the experimental mean and expected values in the vicinity of the peaks and troughs of the first three decay periods were compared. Under initial loads of 0.6, 0.8, and 1.0 Nm, the maximum differences between the expected model and the actual response curves were found to be 10.87%, 9.75%, and 9.84% of the maximum amplitudes, respectively. However, a noticeable error band appeared in the latter part of the actual experimental curves in Figure 4A. This error band is mostly attributed to the dead zone characteristics of the motor.

The experimental results (Figure 4D), including the undamped natural frequency, peak time, and maximum overshoot, were computed, and the relative errors with respect to the target simulated values were extracted to assess the simulation performance of the compliant substrate. The undamped natural frequency was determined to be 5.77 ± 0.05 rad/s, exhibiting a relative error of 0.69% (*n* = 20) compared to the target simulated value of 5.81 rad/s. The actual damping ratio was found to be 0.0507 ± 0.0017 (*n* = 20), with a relative error of 4.52% compared to the target simulated value of 0.0531. Similarly, the actual peak time was determined to be 0.5495 ± 0.0043 s (*n* = 20), yielding a relative error of 1.55% compared to the target simulated value of 0.5411 s. The actual maximum overshoot was measured as 83.71 ± 1.80% (*n* = 20), exhibiting a relative error of 1.06% compared to the target simulated value of 84.61%. Based on the simulated results of the target parameters, the relative errors between the measured results and the target values are all less than 5%. This suggests that, following the calibration and adjustment of device control parameters, the simulation effectively approximates the target substrate characteristics.

For a precise simulation of specific compliant substrates, the methodology proposed in this study relies on a mechanical model corresponding to a particular compliant substrate. Using the mechanical model in [42] as an example, a comparison of the experimental results for an actual palm leaf with those of the theoretical model (Figure 4E) reveals a high degree of alignment in the initial response within the first four decay cycles. This implies that the model used in this study possesses a certain degree of reliability. Furthermore, concerning the simulation of the target parameters in the model, the experimental results demonstrate that the device constructed using the methodology provided in this study can effectively simulate the target substrate. Following the calibration of the mass characteristics of the substrate plane, the relative error between the actual undamped natural frequency of the substrate plane and the target value was less than 1%. The closeness of the undamped natural frequency of the substrate plane to the target value implies a high degree of alignment between the energy conversion periods of the substrate plane response and those of the model. From a more intuitive perspective, the peak time and maximum overshoot of the substrate response were considered key indicators that better assessed the initial response performance. The relative errors between the peak time and maximum overshoot of the substrate response and the target values were both less than 2%, closely resembling the performance of the model. Therefore, the constructed experimental device accurately simulated the target leaf substrate and met the requirements of simulation testing.

### 2.3. Measurement of Contact Force on Leaf-like Compliant Substrates

**Figure 5:**
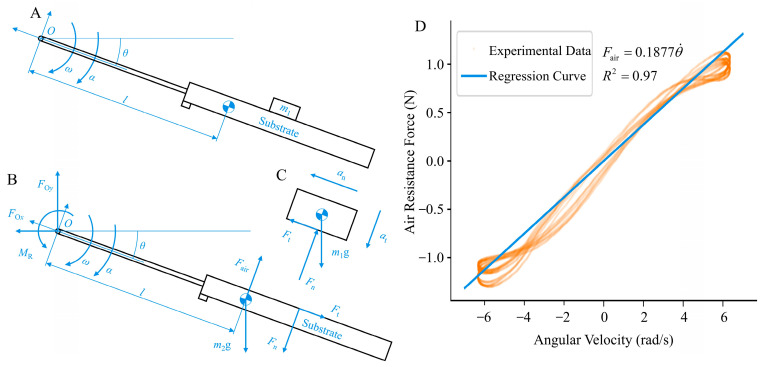
Force analysis and air resistance calibration results of the substrate. (**A**) depicts the contact state between the substrate plane and the object. (**B**,**C**), respectively, illustrate the force distribution on the substrate plane and the object placed on it. (**D**) illustrates the calibration results for air resistance relative to angular velocity. The orange scattered points represent data obtained from multiple periods of unloaded swinging, approximated as a linear relationship and subjected to linear regression, resulting in the blue regression line.

This study employed a method for simultaneous measurements using strain-based multidimensional force and acceleration sensors. The damping control motor stator was rigidly connected to the multidimensional force sensor, facilitating real-time measurements of the actual contact force data. The substrate plane was considered the analytical object in the experimental setup (Figure 5B). According to D’Alembert’s principle, force equations can be established in both the normal and tangential directions on the substrate plane.

(7) $$\left\{\begin{array}{l} F_{t}+m_{2}g\sin\theta +m_{2}\omega^{2}l-F_{Oy}\sin\theta -F_{Ox}\cos\theta =0\\ F_{Oy}\cos\theta -F_{Ox}\sin\theta +m_{2}\alpha l-m_{2}g\cos\theta -F_{n}+F_{\mathrm{air}}=0 \end{array}\right.$$

Rearranging the equation yields

(8) $$\left\{\begin{array}{l} F_{t}=F_{Oy}\sin\theta +F_{Ox}\cos\theta -m_{2}g\sin\theta -m_{2}{\dot{\theta }}^{2}l\\ F_{n}=F_{Oy}\cos\theta -F_{Ox}\sin\theta +m_{2}\ddot{\theta }l-m_{2}g\cos\theta +F_{\mathrm{air}} \end{array}\right.$$

where point *O* is located on the output shaft of the motor, and $F_{Ox}$ and $F_{Oy}$ represent the components of the support force provided by the substrate plane in the absolute coordinate system, both of which can be continuously measured using a multidimensional force sensor. In addition, $\ddot{\theta }$, $\dot{\theta }$, and θ can be obtained from the output of the acceleration sensor.

The air resistance $F_{\mathrm{air}}$ is functionally related to the velocity term [43,44], and its specific relationship can be calibrated through free-swing experiments. In these experiments, the control motor executed reciprocating oscillations with a predetermined amplitude and frequency, ensuring a minimum angular velocity of 5 rad/s to cover the range of angular velocities encountered during the pre-experimental animal motions. A stable operation with continuous analysis over six consecutive cycles was selected from the mechanical data. The variation in air resistance with time was obtained according to the following equation:

(9) $$F_{\mathrm{air}}=F_{Ox}\sin\theta -F_{Oy}\cos\theta +m_{2}g\cos\theta -m_{2}\ddot{\theta }l,$$

Through regression analysis, a functional relationship, $F_{\mathrm{air}}=0.1877$ ($R^{2}=0.97$, *n* = 5430), is established between the air resistance and angular velocity, where the force direction opposes the direction of motion.

The normal contact force $F_{n}$ and tangential contact force $F_{t}$ acting on the animal target can be calculated using Equation (8). The normal contact force $F_{n}$ points towards the animal’s center of mass as positive, indicating a support force from the substrate. When directed away from the center of mass, it is negative, signifying a tendency for the animal to be pulled towards the substrate, suggesting the presence of adhesive effects between the paw and substrate interface. The tangential contact force $F_{t}$ is negative when moving away from the rotational center of the substrate, indicating that, relative to the rotating substrate, the animal experiences a traction force moving away from the substrate. Conversely, when positive, it signifies the animal’s tendency to stabilize on the substrate, resisting the centrifugal force generated by the bending oscillation of the substrate.

### 2.4. Experimental and Analytical Methods

#### 2.4.1. Adhesive Contact Test Calibration

To calibrate the force response pattern of the device for adhesive and non-adhesive contacts, experiments were conducted involving the free-fall impact of standard mass blocks with different masses on the leaf-like compliant substrate plane. The standard mass blocks included both adhesive and non-adhesive types, with each type comprising five different masses, totaling 200 test experiments. Considering that the animals targeted by this experimental device typically fall within the mass range of 50–90 g (such as geckos and tree frogs), three standard blocks with different masses, namely, 50 g, 70 g, and 90 g, were initially selected. To ensure a certain safety margin, the masses of the selected standard blocks should not exceed 80% of the substrate plane mass. Therefore, a standard block with a mass of 140 g was selected as the upper limit, which represents approximately 76% of the mass of the substrate plane. A standard block mass of 40 g was selected as the lower limit.

**Figure 6:**
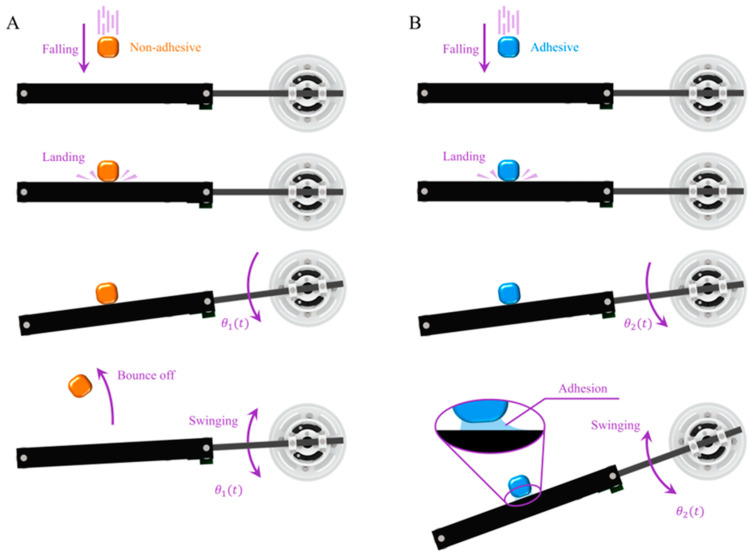
Adhesive contact test calibration process. (**A**,**B**), respectively, depict the landing processes of non-adhesive and adhesive standard mass blocks released from a height onto the substrate plane. In comparison to the rebounding observed with non-adhesive blocks, adhesive standard mass blocks adhere to the substrate plane upon impact, initiating a coupled oscillation with the substrate.

The uniform release height was set to 60 cm, and the initial position of the device was configured with a compliant substrate plane in a horizontal state. In the non-adhesive standard mass block experiment, a standard mass block was released directly above the compliant substrate plane. The mass block made impact contact with the substrate plane and rebounded, and the substrate plane underwent damped oscillation until it rested near its initial position (Figure 6A). In the adhesive standard mass block experiment, the standard mass block was released directly above the compliant substrate plane. It impacted and adhered to the substrate plane. Under the influence of the adhesive characteristics, both the mass block and substrate plane collectively underwent damped oscillations (Figure 6B). This process can be divided into the impact contact stage (from the initiation of contact until the moment of the reverse displacement of the object) and the adhered collective oscillation stage (from the moment of the reverse displacement of the object to the time when the object’s position remains unchanged). The device recorded real-time normal and tangential contact force curves between the mass block and substrate plane during each experimental trial, along with the corresponding collision behavior states. The extracted parameters include the maximum values of the normal and tangential contact forces as well as the frictional (adhesive) coefficients during the adhesive oscillation contact process.

The adhesive standard block oscillated with the substrate plane after contact with it and maintained a relatively certain motion state after impact. Therefore, to verify the force measurement accuracy of the device, the motion data of the adhesive standard block recorded by a motion capture system was used to estimate the theoretical values of the mean impact force between the standard block and the substrate plane during the initial impact process. The calculation period for the mean impact force started from the beginning of the impact until the adhesive standard block and the substrate plane reached their shared maximum velocity. The mean acceleration was determined by analyzing the velocity variation of the adhesive standard block during this period in each experiment, thereby obtaining the theoretical estimated values of the mean impact force. A comparison was made between these theoretically estimated values and the measured values of the mean impact force during the corresponding period. Based on this, the reliability of the force measurement device was evaluated.

#### 2.4.2. Animal Landing and Takeoff Experiment

Two specimens each of two distinct adhesive animals, *Gekko gecko* and *Zhangixalus dennysi*, were selected for landing and takeoff experiments on a compliant substrate system to validate the testing performance of the device with respect to their motion behavior and contact forces on compliant substrates. The *Gekko gecko* specimens had masses of 91.5 and 74.7 g, snout–vent lengths (SVLs) of 16.2 and 15.3 cm, and overall lengths of 31.3 and 26.7 cm, respectively. The two *Zhangixalus dennysi* tree frogs had masses of 50.5 and 48.1 g and SVLs of 8.8 and 8.5 cm, respectively. The animals were maintained in environments with temperatures ranging from 25 °C to 28 °C, humidity between 60% and 80%, and half-day lighting. They were fed every two days with live insects and provided with fresh water daily. This experimental protocol adhered to the guidelines for handling animals in behavioral studies established by the ASAB, approved by the Jiangsu Experimental Animal Science Association, and conducted in accordance with the regulations of Chinese Experimental Animal Management. During the experiments, the initial position of the compliant substrate plane was horizontal, with a sponge cushion placed below it to ensure the safety of the experimental animals during unexpected events.

In the experiments investigating the adhesive landing behavior and contact force on the compliant substrate, the height difference between the animal’s release position and the initial position of the compliant substrate plane was set at 60 cm. The release position was determined using the intersection point of a pair of orthogonal infrared light beams installed on a support frame to ensure consistency in the release position of the animal. Additionally, all the animals were released with their ventral sides facing downward. For force measurement during takeoff, animals were induced to jump off the compliant substrate plane by placing a dimly lit trap box in front of the substrate. The bottom plane of the trap box was positioned 8 cm below the initial position of the substrate plane, at a horizontal distance of 30 cm.

The device recorded real-time normal and tangential contact force curves between the animals and the compliant substrate plane during each landing and takeoff experiment. Considering individual variations, the obtained contact force data were normalized based on the respective body weights (BWs) of the animals. The force curves were compared with the calibration results from the adhesive contact tests to assess the adhesive biomechanical performance of the animals during animal–substrate contact. Statistical analyses were conducted on the maximum values of the normal and tangential forces, as well as the friction (adhesive) coefficient, $\mu_{v}$, obtained in each experiment. The analysis also incorporated high-speed camera footage to capture the animals’ movement behaviors for a comprehensive understanding of the force curves.

## 3. Results and Discussion

### 3.1. Adhesive Contact Test Calibration Results

**Figure 7:**
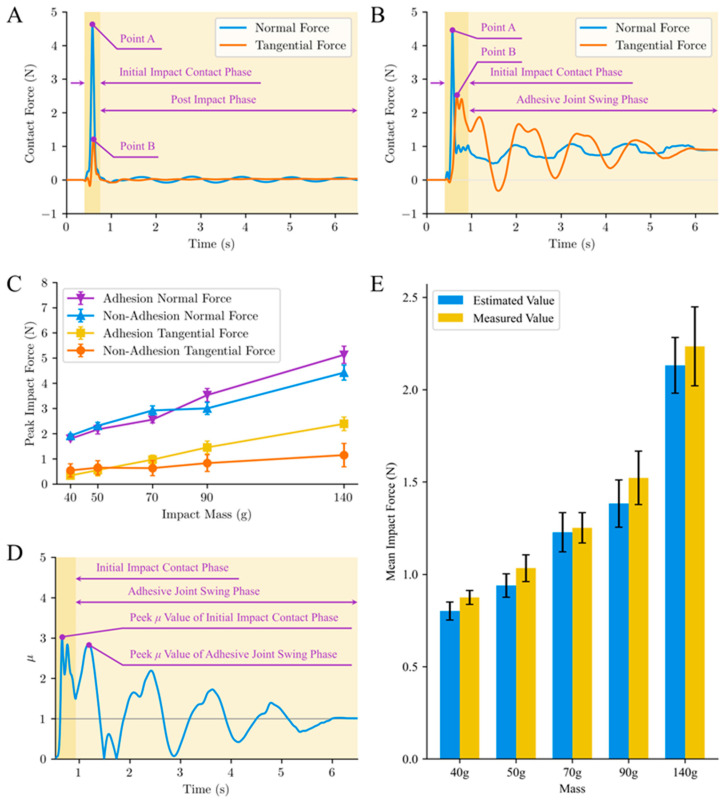
Adhesive contact test calibration results. (**A**,**B**) depict the contact force curves from a specific experiment with non-adhesive and adhesive blocks, each with a mass of 140 g, respectively. “In these figures, Points A and B represent the peak values of normal and tangential contact forces, respectively. (**C**) provides a statistical analysis of the maximum contact force results. Significant differences (*p* < 0.001) in the maximum contact force values in both normal and tangential directions are observed when the mass exceeds 50 g for both adhesive and non-adhesive conditions. (**D**) illustrates the temporal variation of the frictional (adhesive) coefficient $\mu_{V}$ corresponding to the contact force curve in (**B**) (i.e., tangential force/normal force). The symbols for tangential contact force only represent directional differences and have undergone absolute value processing. Due to the significant influence of measurement errors on the coefficient of friction $\mu_{V}$ when both normal and tangential contact forces are relatively small, the corresponding $\mu_{V}$ values lack meaningful research significance. The zero-crossing point of the stable rising phase of the tangential contact force is thus designated as the starting recording point for $\mu_{V}$. (**E**) shows the comparison between the estimated values and the measured values of mean impact forces of the adhesive standard block during the initial impact.

The dynamic contact behaviors and mechanical performances of the adhesive and non-adhesive standard mass blocks exhibited evident differences (Figure 7A,B). In the initial contact state (from initial contact to the moment of reverse displacement), the non-adhesive calibration blocks experienced distinct impact collisions with the substrate, manifested as peaks in the force waveform (Figure 7A). As the mass increased from 40 g to 140 g, the peak normal force of non-adhesive standard blocks gradually increased from 2 N to 5 N, and the tangential adhesive force increased from 0.3 N to 1.1 N (Figure 7C). In contrast to previous measurement devices designed for compliant substrates [10,11,26], the contact force measured by this device did not exhibit significant fluctuations with the oscillation of the substrate when the non-adhesive standard block detached from the compliant substrate. This difference is attributed to the device’s utilization of a combined force and acceleration sensor measurement approach for dynamic contact processes on compliant substrates. The compensatory effect of inertial forces during substrate motion was achieved, ensuring that the contact force curve promptly returned to near zero when the non-adhesive standard block detached from the compliant substrate. The moment of contact for the adhesive standard mass block was similar to that for the non-adhesive standard mass block, showing an initial contact impact effect (Figure 7B). With an increase in the mass of the standard block, both the peak normal and tangential forces exhibited an ascending trend. The tangential contact force between the adhesive standard mass block and substrate increased notably faster than that for the non-adhesive standard mass block. When the mass reached 140 g, the peak tangential contact force between the adhesive standard mass block and substrate exceeded 2 N, attaining nearly twice the value for the non-adhesive standard block. This can be attributed to the relative tangential adhesive sliding tendency induced by the impact force, which occurs in conjunction with the oscillation of the compliant substrate.

During the impact process between the adhesive standard block and the substrate plane, the comparison between the theoretical estimated values and the actual measured values of the mean impact forces is illustrated in Figure 7E. In the experiments of five adhesive standard blocks with different masses, the relative errors between the estimated and the device-measured values of the mean impact force during the corresponding periods were not more than 10%. Furthermore, when the mass of the standard block was greater than 50 g, there was no significant difference between the estimated and measured values (*p* < 0.001). These results indicate that the device built in this study exhibits reliable measurement capabilities for the actual contact force in dynamic contact processes.

Although various biomimetic manufacturing methods have successfully produced micro-/nanostructures with high adhesive forces in dry adhesion materials, the lack of dynamic adhesive performance, such as the response of normal and tangential adhesion properties to preload loading rates, limits their engineering applications. Biomimetic dry adhesive surfaces exhibit viscoelastic characteristics [45], and the constitutive relationship between viscoelasticity exhibits nonlinear and time-dependent features, resulting in distinct outcomes under dynamic and static loads. After the initial contact state is concluded, the non-adhesive standard block rebounds away from the compliant substrate plane owing to collision, while the adhesive standard block encapsulated with the biomimetic dry adhesion material maintains contact with the object owing to the adhesive effect. With the oscillation of the compliant substrate, the normal and tangential contact forces exhibited periodic attenuating fluctuations, and the frictional (adhesive) coefficient $\mu_{v}$ > 1 (i.e., tangential force/normal force > 1) demonstrated noticeable frictional adhesion [35]. The adhesive force was only 2.4 N, which is considerably less than the adhesive force under static loading, being only 0.3 times the static adhesive force [46]. This is consistent with previous findings from tests of the normal and tangential adhesive forces of adhesive materials under static and dynamic loads, indicating that the dynamic adhesive force of the adhesive materials is weaker than the static adhesive force [47,48]. The compliant substrate, characterized by its high dynamic features, further diminishes the adhesive performance of dry adhesive contact interfaces under dynamic contact conditions. This underscores the necessity of designing bioinspired attachment mechanisms on compliant substrates to enhance the effective contact force and area of dynamic contact instant adhesive end effectors.

### 3.2. Contact Forces of Adhesion Landing/Takeoff from Leaf-like Compliant Substrates

#### 3.2.1. Landing

**Figure 8:**
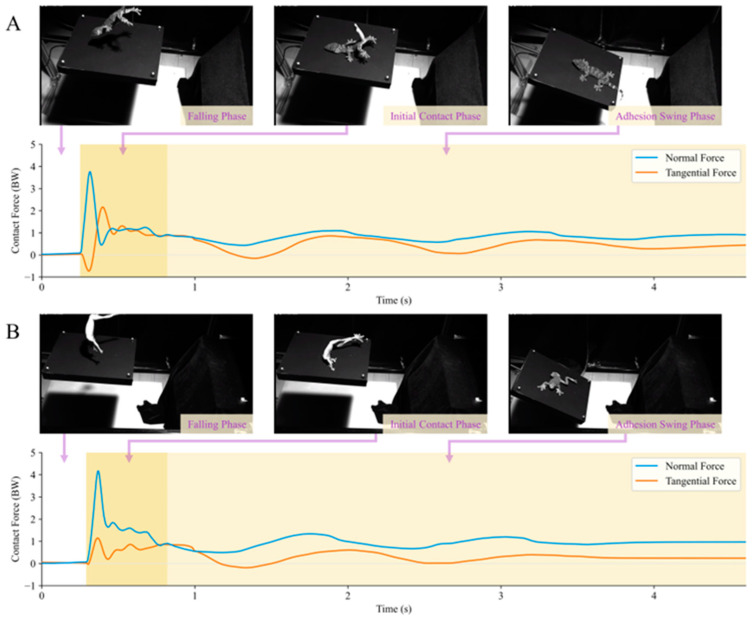
Contact force behavior of adhesive animals landing on the compliant substrate. (**A**,**B**) depict the landing contact force behaviors of the gecko and tree frog on the substrate plane, respectively. These are divided into three main phases: the falling phase, the initial contact phase (from the moment of contact until the reversal of animal displacement), and the adhesive swing phase (from the initiation of animal displacement reversal until the point where the animal’s position ceases to change).

On compliant substrates, both the geckos and tree frogs exhibited analogous processes during adhesive landing (Figure 8). Upon release, the animals underwent a falling phase and made contact with the substrate plane, subsequently landing on the substrate surface under the influence of adhesive forces after experiencing an initial impact. To prevent slipping from the substrate plane, the animals engaged in a damping oscillation along with the compliant substrate, facilitated by frictional adhesive forces, until both the animal and substrate plane stopped moving. The phase before an animal makes contact with the substrate plane during a fall is referred to as the falling phase. The period from the initial contact between the animal and substrate plane until a reversal in animal displacement is observed is denoted as the initial contact phase. The interval from the reversal of animal displacement until no further changes in the animal’s position occur is identified as the adhesive swing phase.

In the contact force behavior measurement of *Gekko gecko* in the compliant substrate adhesion landing behavior experiment (Figure 8A), the tangential contact force peak in the initial contact phase was approximately 2.60 ± 0.49 BW (*n* = 10), whereas the instantaneous normal contact peak force was 2.86 ± 0.65 BW (*n* = 10). In this phase, the gecko exhibited a peak frictional adhesive coefficient $\mu_{v}$ of 3.37 ± 1.30 (*n* = 10). During the adhesive oscillation phase, the peak tangential adhesive force was 1.16 ± 0.14 BW (*n* = 10), and the gecko exhibited a peak $\mu_{v}$ value of 1.93 ± 0.70 (*n* = 10). In the adhesive landing behavior experiment, in the contact force measurements of *Zhangixalus dennysi* (Figure 8B) during the initial contact phase, the instantaneous tangential contact force between the frog and the substrate was approximately 1.26 ± 0.42 BW (*n* = 10), whereas the instantaneous normal contact force was 3.55 ± 0.59 BW (*n* = 10). In this phase, the tree frog demonstrated a peak $\mu_{v}$ value of 0.90 ± 0.19 (*n* = 10). During the adhesive oscillation phase, the adhesive force peak was 1.00 ± 0.10 BW (*n* = 10), and the peak $\mu_{v}$ value was 1.02 ± 0.16 (*n* = 10).

As shown by the aforementioned landing behavior experiments of adhesive animals, both the gecko and tree frog exhibit contact force curves similar to those of the adhesive standard mass block in Figure 7B. Furthermore, according to the landing contact force curves, both animals exhibited frictional adhesive coefficients $\mu_{v}$ > 1, similar to the situation depicted in Figure 7B. Therefore, the frictional adhesive properties were effectively measured. Additionally, in this substrate environment, the peak frictional adhesive coefficient of the gecko during the initial contact phase was slightly higher than that during the adhesive swing phase, whereas this distinction was not prominently observed in the case of the tree frog. This discrepancy may arise from differences in adhesive mechanisms and distribution between the two adhesive animals. In terms of actual frictional adhesive force performance, compared to theoretical estimates from previous studies, the gecko’s maximum tangential adhesive force observed in the experiment was only approximately 3.2 times its body weight, approximately 30% to 40% of the theoretical value [16]. Similarly, the tree frog’s maximum tangential adhesive force observed in the experiment was only approximately 2.5 times its body weight, roughly 20% of the theoretical value [49]. This suggests that in the actual process of adhesive motion on compliant substrates, these adhesive animals may employ unique strategies to regulate their adhesive performance, allowing for successful landing in a highly safe margin-oriented manner. However, it is crucial to acknowledge the potential influence of factors, such as surface contact characteristics and substrate morphology states, in this context.

#### 3.2.2. Takeoff

**Figure 9:**
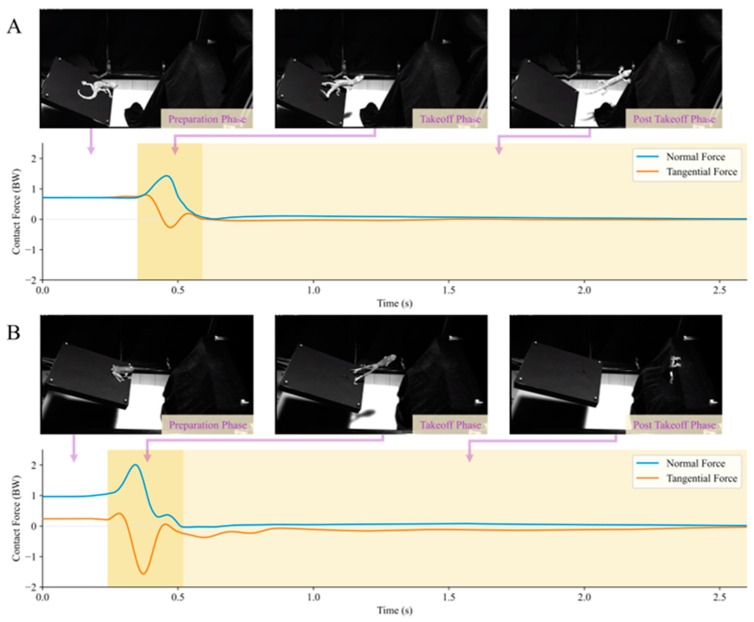
Contact force behavior of adhesive animal takeoff from the compliant substrate. (**A**,**B**) depict the takeoff contact force behaviors of the gecko and tree frog on the substrate plane, respectively. Here, these are divided into three main phases: the preparation phase, the takeoff phase (from the initiation of takeoff until the animal completely detaches from the substrate plane), and the post-takeoff phase (after the animal has completely detached from the substrate plane).

Both adhesive animals exhibited similar motion processes during adhesive jumping on the compliant substrate (Figure 9). After determining the target landing position, the animals propelled the substrate with their limbs and moved their bodies towards that position under a reaction force. During this process, the animal’s body gradually moved away from the substrate plane until it completely lost contact with the substrate. The period before takeoff is referred to as the preparation phase; the process from the beginning of takeoff until the animal completely detaches from the substrate plane is the takeoff phase; and the stage after the animal completely detaches from the substrate plane is termed the post-takeoff phase.

In the adhesive takeoff behavior contact force measurement experiment with geckos, the device recorded its maximum normal contact force on the substrate as 1.13 ± 0.31 BW (*n* = 6) and the maximum tangential contact force as 1.19 ± 0.55 BW (*n* = 6). During their takeoff phase, the geckos exhibited a peak frictional adhesive coefficient $\mu_{v}$ of 2.16 ± 1.52 (*n* = 6). In the adhesive takeoff behavior contact force measurement experiment with tree frogs, the device recorded its maximum normal contact force on the substrate as 1.50 ± 0.26 BW (*n* = 10) and the maximum tangential contact force as 1.00 ± 0.31 BW (*n* = 10). During their takeoff phase, the tree frogs exhibited a peak $\mu_{v}$ of 1.79 ± 0.44 (*n* = 10).

In the aforementioned results, both adhesive animals exhibited a frictional adhesive coefficient of $\mu_{v}$ > 1 during takeoff. This indicates that during takeoff on the compliant substrate, frictional adhesive forces, in addition to the normal force provided by the substrate plane, also play a significant role in propelling the animal’s bodies through this gap-crossing behavior. Moreover, the contact force curves of the takeoff behavior in Figure 9 show that the contact forces of both the gecko and tree frogs, as measured by the experimental setup, returned to near zero after detachment. This phenomenon is similar to that in the post-impact phase depicted in Figure 7A, and the animals were no longer in adhesive contact with the compliant substrate in the post-takeoff phase.

### 3.3. Performance Comparisons and Limitations

Mechanical studies related to compliant substrates are of unique complexity, resulting in slow progress. Among the existing methods/devices for measuring the interaction force between the animal and the compliant substrate, when an external object is in contact with the substrate (e.g., the elastic collision in Figure 6A and the animal’s jumping behavior in Figure 9), force variations at this time can be obtained. However, after the interface contact disappears, force data signals with periodically decaying fluctuations are still obtained [26], regarded as ordinary fluctuations of force signals induced by the oscillation of the compliant substrate. The appearance of reaction forces is based on the existence of contact interactions between the animal and the substrate environment. When the animal is completely detached from the substrate, there will be no interaction force between them. This means that the contact forces obtained are coupled with the oscillatory inertial forces of the compliant substrate itself under the effect of the impact force. In the device shown in this paper, this oscillatory inertial force is quantitatively measured in real time using a sensitive acceleration sensor mounted on the compliant substrate. The results of the impact experiment with non-adhesive standard mass blocks (Figure 7A) and the animal takeoff experiment (Figure 9) in this paper show that when the oscillatory force is accurately characterized, the force data return to near 0 without significant numerical changes when the contact disappears, validating the inferences made in this paper.

In this study, the influence of the oscillatory inertial force of compliant substrates and the control of the damping characteristics are considered, effectively improving the accuracy of the contact force measurement. However, the simulation of the target substrate mainly focuses on the foliage bending motion, which is unidirectional compliance. Like previous theoretical [16] and experimental [10,11,26] studies, this simplification lacks sufficient characterization of the complexity of compliant substrates in nature. Twisting and local compliance [42] need to be introduced into further studies of leaf-like compliant substrates. In addition, as mentioned previously, the simulation accuracy of compliance in the bending direction in this study is also limited by the motor dead zone characteristics (Figure 4A) and the substrate mechanics model (Figure 4E). Improvement of these conditions requires the future development of advanced motor technologies and further research on the mechanical models of different complex substrate environments.

Table 1 shows a performance comparison of the device presented in this paper with previous similar compliant substrate force measurement devices. Although this study is limited to single-direction compliance in the simulation of the compliant substrate, the servo motors in this paper are used to achieve a parameterized and controllable stiffness–damping adjustment of the compliant substrate. This is in contrast to previous studies on primates [11,26] and tree frogs [10], which only adjusted the stiffness characteristics. Regarding force measurements, the devices used for primate research [26] and tree frog research [10] only measured the normal reaction force. However, many arboreal species exhibit impressive locomotor behaviors in compliant substrate environments, often utilizing frictional adhesive forces [16]. The measurement of tangential force by the device in this research is crucial for studying animal mechanics on compliant substrates, particularly for adhesive animals (Figure 8 and Figure 9). This device possesses relatively comprehensive performance (addressing the effect of oscillatory inertial force, having controllable stiffness–damping characteristics, etc.), which provides an experimental technique for the study of animal locomotion mechanics on compliant substrates.

## 4. Conclusions

This study employed a servomotor control system incorporating a stiffness–damping model to achieve a more precise and reliable simulation of compliant substrates resembling leaf veins. The relative error of the characteristic parameters between the leaf-like compliant substrate and simulated target leaf veins was consistently less than 5%. Acceleration and force sensors were integrated with the leaf-like compliant substrate, eliminating the effect of inertial force coupling on the accuracy of contact force measurements that existed in previous studies. Quantitative calibration of the adhesive contact process was conducted through comparative experiments involving non-adhesive and adhesive calibration blocks. Within this calibration process, the device verified that the relative error between actual measured and theoretical estimated values for the adhesive impact contact was not greater than 10%. In real-time measurements of adhesion contact force in the landing/takeoff of adhesive animals on the compliant substrate, it was found that the maximum value of adhesion force was only 20~40% of the theoretical capacity value. The device could be used to further explore the possible mechanisms behind adhesion regulation in the future. The improved accuracy of force measurement on compliant substrates in this study can facilitate the exploration of animal locomotor stability in complex substrate environments and promote the study of animal compound locomotor behavior. The research outcomes are also expected to be applied in robotics research and can be used as an experimental testing platform to optimize the stability of robotic motion.

The leaf-like compliant substrate provided in this study can simulate the oscillation characteristics of the contact points on the main vein of leaves with different compliance under external loads by adjusting the stiffness–damping characteristics of the servomotor and the length of the cantilevered structure. However, it is limited to measuring the overall contact force between the animal and the substrate plane. Based on the method principles provided in this study, the discrete multi-cantilever array model can be constructed in the future to improve the synchronized testing of adhesion contact force at multiple points. Moreover, the mechanical response model of the target substrate can be further optimized and extended to create more accurate and diversified simulated substrates. This will help promote future research on the mechanical measurement system of full-space controllable compliant substrates.

## Figures and Tables

**Table 1:** Comparison of different research devices for compliant substrates.

Comparison Items	Device for Primate Research [26]	Device for Gibbon Research [11]	Device for Tree Frog Research [10]	Device in This Research
Control of substrate compliance	Stiffness	Stiffness	Stiffness	Stiffness, damping
Measurement of contact forces	Normal force	Normal force, tangential force	Normal force	Normal force, tangential force
Consideration of inertial forces due to compliant substrate oscillation	No	No	No	Yes

## Data Availability

The data generated and/or analyzed in the current study are not publicly available for legal/ethical reasons but are available from the corresponding author upon reasonable request.
